# Case Report: Intramammary lymph node metastasis of an unknown primary, probably occult breast, undifferentiated carcinoma

**DOI:** 10.12688/f1000research.11065.1

**Published:** 2017-03-14

**Authors:** Zacharoula Sidiropoulou, Félix Adélia, Isabela Gil, Tobias Teles, Claudia Santos, Lucilia Monteiro

**Affiliations:** 1 General Surgery Department, Breast Unit, Hospital São Francisco Xavier-CHLO, Lisbon, 1449-005, Portugal; 2 Medical Oncology Department, Breast Unit, Hospital São Francisco Xavier-CHLO, Lisbon, 1449-005, Portugal; 3 General Surgery Department, Hospital São Francisco Xavier-CHLO, Lisbon, 1449-005, Portugal; 4 Pathology Department, Breast Unit, Hospital São Francisco Xavier-CHLO, Lisbon, 1449-005, Portugal

**Keywords:** Breast cancer, occult breast cancer, intramammary lymph node metastasis, multidisciplinary approach

## Abstract

Little is known about the clinical importance of intramammary lymph node metastasis of breast cancer, even though it is not rare. In the present paper, the authors present an unusual, rare case of an intramammary lymph node metastasis of an unknown primary, probably occult breast cancer, and its management. The patient was submitted to various staging exams and surgical procedures and a definitive diagnosis was not established. From a multidisciplinary context, it was assumed that the patient had a breast triple negative primary with axillary involvement. This decision lead to adjuvant chemo and radiotherapy. Challenging cases like the one described here, should always be managed within the multidisciplinary team context and recorded in the institution’s database.

## Background

Intramammary lymph node metastasis is an unknown in everyday clinical practice and very little is known about its importance.

## Case presentation

A woman, 33 years old, from Goa (India) presented to our consultation for a palpable mass on the upper external quadrant of the right breast. The patient had no personal relevant history. Menarche had occurred at 15 years with regular menses of 4/26 days, G0P0, without anticonceptional pills use, and no drug or alcohol abuse. The patient’s family history showed that the mother passed away at 40 years old with metastatic (brain) breast cancer and her maternal uncle was deceased at the age of 45 from esophageal cancer.

## Investigations

**Figure 1.**
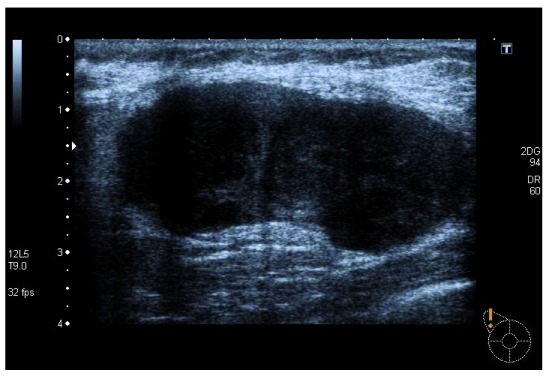
Ultrasonography of nodular formation at the upper external quadrant of the right breast.

The patient had already undergone ultrasound and bilateral breast mammography that reported the ‘presence of nodular multiloculated formation at the upper external quadrant of the right breast with 3 cm of diameter, probably corresponding to inflammatory/infectious lymph node’ ( Figure 1).

On clinical observation, voluminous breast with grade III ptosis and a palpable solid mass was observed. It was an irregular mass of approximately 4 cm on the upper external right breast quadrant, not adherent to the skin or to the pectoralis muscle. The patient was submitted to an ultrasound guided fine-needle aspiration biopsy (FNAB) that reported ‘fragments of lymph node with poorly differentiated neoplastic infiltration. Presence of epithelioid neoplastic cells positive for AE1/E3 and negative for CK20, CEA, vimentin, protein S100, P63, CD56, TTF-1, GCDFP-15, estrogen receptors. Conclusion: lymph node metastasis of poorly differentiated carcinoma of unknown primary origin’.

**Figure 2.**
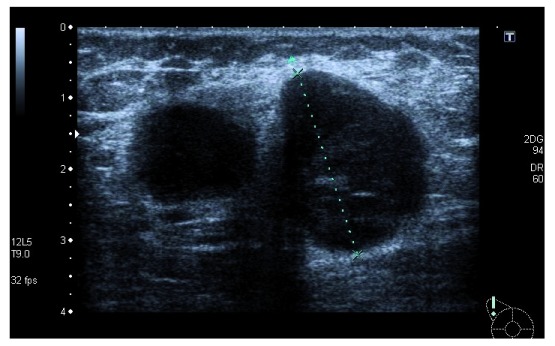
Ultrasound second look and fine-needle aspirate biopsy of the second lesion.

The patient underwent a magnetic resonance imaging scan in which there was detected an additional 17mm lesion (BI-RADS-5) adjacent posterior to the lymph nodal mass previously detected, which was submitted to an ultrasound second look and FNAB ( Figure 2). In this biopsy, no neoplastic tissue was identified, and the results reported ‘mammary gland fragments with inflammatory process, no isolated epithelial cells identified after IHC with CK8/18’.

Consequently, the decision of the multidisciplinary team was to perform complementary studies (upper gastroscopy, otorhinolaryngological consultation, dermatology consultation, thoracic-abdominal-pelvic tomography, and full analytics with tumor markers). All the complementary studies were negative. Therefore, the multidisciplinary team decided that ‘the patient to be proposed for lumpectomy with axillary lymphadenectomy’ with a PET-TC scan positive only for the mass to the upper external quadrant of the right breast. The patient was submitted to lumpectomy on oncoplastic pattern, followed by axillary dissection level II, and was discharged without any complication on the third post-operative day.

**Figure 3.**
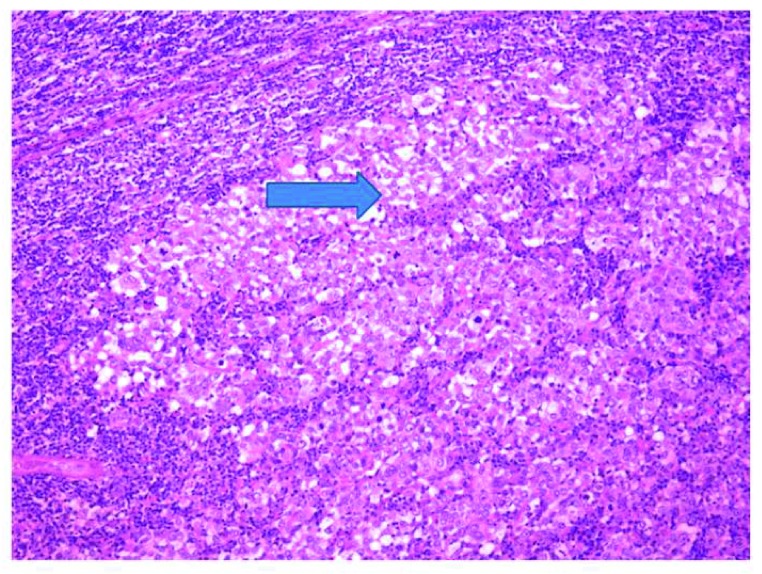
Metastatic lymph node agglomerate.

The anatomopathology report of the surgical specimen stated that the ‘lumpectomy specimen constituted of skin, adipose tissue and mammary tissue where there exists a nodule, well delimited, white, with posterior margin of 1mm, consisting of a lymph node agglomerate with poorly differentiated metastasis with CK7 positive and rare CD56 positive cells, focally positive for EMA’ ( Figure 3). In addition, the ‘lymphadenectomy specimen [had] 15 reactive, free of metastasis, lymph nodes’.

A second pathology review of the lumpectomy specimen (external to our institution), indicated that the excised nodule consists of five lymph nodes as an agglomerate with histology of an undifferentiated metastasis, of a probable triple negative of mammary origin primary tumor. Therefore, the multidisciplinary team decided to propose the patient for total mastectomy, which was performed and the anatomopathological report showed neither abnormalities, nor the presence of neoplastic tissue in the remaining breast.

## Treatment

In an adjuvant setting, the patient was administered with the TAC chemotherapy protocol (docetaxel 75 mg/m2, doxorubicin 50mg/m2 and cyclophosphamide 500mg/m2, every 21 days, accompanied with pegfilgrastim) and successfully completed 6 cycles. The patient later received standard thoracic and lymphatic chain radiotherapy (50 Gy in 25 fractions over 5 weeks and boost to the tumor bed).

BRCA 1 and 2 genetics were negative.

## Outcome and follow-up

The patient is currently in remission and had an uneventful follow-up at the Medical Oncology and Senology Department at our institution. According to our protocol, the patient undergoes clinical observation every three months accompanied by laboratory full set analysis (tumour markers included) and an annual breast imaging

## Discussion

Very little is known about the clinical importance of intramammary lymph node metastasis of breast cancer, even if they are not a rare site for metastasis. However, it is believed that metastasis to intramammary lymph nodes is an independent factor of poor prognosis for breast cancer patients ^1, 2^.

In a Pubmed search between 1900 and 2016, there is only one paper concerning metastatic intramammary lymph nodes as the primary presenting sign of occult breast cancer, which describes two cases ^3^. The cases presented by Kouskos *et al* ^3^ have some histological differences from the present one (for example, estrogen receptor positivity, axillary lymph node involvement, and late presentation of the primary breast tumour), and also the late appearance of the primary breast tumour. In our case and up until now, we have never detected a primary breast tumor. Similarly to our case, the other cases required an extensive complementary study of the patient.

Our decision to treat the patient as a triple negative breast cancer patient with axillary metastatic involvement was been based on the histopathological suspicion of a breast-like primary site and the patient’s strong family history (1 ^st^ degree familiar with breast cancer at <40 years old age).

In conclusion, intramammary lymph node metastasis requires a challenging workup and there is an urgent need to clarify its importance. Breast cancer patients should always undergo treatment in a multidisciplinary context. Being an extremely rare event, the one described here, good medical practice imposes a broad discussion among the various specialities that only can be achieved in the multidisciplinary setting. Decisions about treatment strategies to be offered are vast and should be patient centred.

## Take home messages

Intramammary lymph node metastasis requires challenging workup

There is urgent need to clarify its importance

Breast Cancer patients should always undergo treatment in multidisciplinary context

## Consent

Written informed consent from the patient has been obtained for the publication of this manuscript.
