# Supplementary material for: Case Report: Intramammary lymph node metastasis of an unknown primary, probably occult breast, undifferentiated carcinoma
Source: F1000Res. 2017 Mar 14;6:265. [Version 1] doi: 10.12688/f1000research.11065.1 (PMC5499792; doi:10.12688/f1000research.11065.1)
Supplement: Peer review history

# Peer review: Referee response for version 1

**DOI:** 10.5256/f1000research.11933.r23354

**Published:** 2017-06-30

**Authors:** Kenji Gonda

**Affiliations:** 1 Division of Breast and Endocrine Surgery, Department of Surgery, Nihon University School of Medicine, Tokyo, Japan

The authors described that Intramammary lymph node metastasis of an unknown primary, probably occult breast, undifferentiated carcinoma. These findings in this manuscript are interesting, and this manuscript is worthy of indexing. There are some problems should be resolved before publishing. 

1. What is the TNM classification for staging of this breast cancer patient?
2. Do you think that this ectopic breast tissue may be accessory breast cancer?
3. You should reveal breast pathological diagnosis of the lumpectomy specimen, for example, papillo-tubular carcinoma or scirrhous carcinoma with mammary gland.
4. What is the result of the human epidermal growth factor receptor 2 (HER-2/neu) and Ki-67 marker?
5. You should refer to an article of Egan, because intramammary lymph node metastases in the breast were reported for the first time in the world by Egan and McSweeney in 1983 ^1^.
6. Are additional ancillary studies, including immunostainings, beneficial to evaluate site of origin (in case of this tumor is CD10 focally positive)?
7. What are the second line therapy options for this rare pathology, when this patient will have recurrence of breast cancer?
8. What is the role of family history and its impact on therapy?

I have read this submission. I believe that I have an appropriate level of expertise to confirm that it is of an acceptable scientific standard.

## References

[1] EganRLMcSweeneyMB: Intramammary lymph nodes. *Cancer*.1983;51(10) :1838-42 629949810.1002/1097-0142(19830515)51:10<1838::aid-cncr2820511014>3.0.co;2-8

# Peer review: Referee response for version 1

**DOI:** 10.5256/f1000research.11933.r21962

**Published:** 2017-05-30

**Authors:** Ramesh Omranipour

**Affiliations:** 1 Division of Surgical Oncology, Cancer Institute, Tehran University of Medical Sciences, Tehran, Iran

It is better to added the images of MRI and PET scan of the patient.

 The discussion is too brief, although this presentation for intramammary lymph node is rare but there are many reports in the literature about the clinical importance and prognostic value of involved intramammary lymph node.

I have read this submission. I believe that I have an appropriate level of expertise to confirm that it is of an acceptable scientific standard, however I have significant reservations, as outlined above.

# Response

**Published:** 2017-06-07

**Authors:** Zacharoula Sidiropoulou

**Affiliations:** Hospital São Francisco Xavier CHLO Lisbon, Portugal

Dear Colleague,

First of all thank you for your comments.

As a reply:

"Better to added the images of MRI and PET scan of the patient" 

- Unfortunately we are not in possession of this imaging

"Discussion is too brief, although this presentation for intramammary lymph node is rare but there are many reports in the literature about the clinical importance and prognostic value of involved intramammary lymph node."

- Hereby our intention was just to report this specific unusual case, our first submission was more extended but afterwards we decided to limit to the presentation and not to proceed to a literature review of intramammary lymph node involvement in  known,diagnosed breast cancer

We hope our answer meet your kind and helpful comments

Once more thank you for the review and we wait your feedback.

# Peer review: Referee response for version 1

**DOI:** 10.5256/f1000research.11933.r21961

**Published:** 2017-04-24

**Authors:** Sergi Vidal-Sicart, Immaculada Alonso

**Affiliations:** 1 Nuclear Medicine Department, Hospital Clinic Barcelona, Barcelona, Spain; 2 Gynaecology and Obstetrics Department, Hospital Clinic Barcelona, Barcelona, Spain

It is a well-written paper concerning an infrequent case of intramammary metastases without a known primary tumor.

 The case description is adequate and nicely presented. We only have to add some minor requirements to the authors.

- It could be adequate to add the MR and PET images demonstrating their findings.
- Did the authors consider that carboplatin could be added to the treatment. It seems that this agent offers good results in TN breast cancer.
- Finally, do you consider to expand the genetic study, even with a negative BCRA, due to a possibility to express other gens like PALB2?

We have read this submission. We believe that we have an appropriate level of expertise to confirm that it is of an acceptable scientific standard.
